# Molecular Principles of Intrauterine Growth Restriction in Plasmodium Falciparum Infection

**DOI:** 10.3389/fendo.2019.00098

**Published:** 2019-03-01

**Authors:** Johanna Seitz, Diana Maria Morales-Prieto, Rodolfo R. Favaro, Henning Schneider, Udo Rudolf Markert

**Affiliations:** ^1^Placenta Lab, Department of Obstetrics, Jena University Hospital, Jena, Germany; ^2^Institute of Biochemistry and Molecular Medicine, University of Bern, Bern, Switzerland; ^3^Department of Obstetrics and Gynecology, Inselspital, Bern University Hospital, University of Bern, Bern, Switzerland

**Keywords:** malaria, plasmodium, pregnancy, placenta, intrauterine growth restriction, small for gestational age, anemia

## Abstract

Malaria in pregnancy still constitutes a particular medical challenge in tropical and subtropical regions. Of the five *Plasmodium* species that are pathogenic to humans, infection with *Plasmodium falciparum* leads to fulminant progression of the disease with massive impact on pregnancy. Severe anemia of the mother, miscarriage, stillbirth, preterm delivery and intrauterine growth restriction (IUGR) with reduced birth weight are frequent complications that lead to more than 10,000 maternal and 200,000 perinatal deaths annually in sub-Saharan Africa alone. *P. falciparum* can adhere to the placenta via the expression of the surface antigen VAR2CSA, which leads to sequestration of infected erythrocytes in the intervillous space. This process induces a placental inflammation with involvement of immune cells and humoral factors. Especially, monocytes get activated and change the release of soluble mediators, including a variety of cytokines. This proinflammatory environment contributes to disorders of angiogenesis, blood flow, autophagy, and nutrient transport in the placenta and erythropoiesis. Collectively, they impair placental functions and, consequently, fetal growth. The discovery that women in endemic regions develop a certain immunity against VAR2CSA-expressing parasites with increasing number of pregnancies has redefined the understanding of malaria in pregnancy and offers strategies for the development of vaccines. The following review gives an overview of molecular processes in *P. falciparum* infection in pregnancy which may be involved in the development of IUGR.

## Introduction

Pregnant women are more susceptible to infection with *Plasmodium falciparum* and present a more severe form of the disease than non-pregnant women (1). The probability of suffering from severe malaria infections is three times higher—with a mortality rate of up to 50% (2, 3). Other complications involve severe anemia, cerebral malaria, and massive pregnancy disorders (4). The increased susceptibility is attributed to two main factors: firstly, physiological processes during pregnancy, such as the altered hormone constellation with suppression of certain immune reactions, and increased body temperature, which makes pregnant women more attractive to Anopheles mosquitoes (5, 6); secondly, the sequestration of *P. falciparum*-infected erythrocytes in the placenta (7).

In placental malaria, *P. falciparum* expresses a special Plasmodium falciparum erythrocyte membrane protein 1 (PfEMP-1), the VAR2CSA antigen, which can bind to chondroitin sulfate A (CSA) produced by trophoblast cells. This interaction promotes the retention of parasites in the intervillous space triggering an inflammatory reaction known as intervillitis. Women in endemic regions often have developed humoral immunity reflected by the production of antibodies against different PfEMP-1 expressing *P. falciparum* strains. However, as the VAR2CSA appears only in pregnancy, primiparous women have no antibodies against this antigen yet, and again, are at high risk for a new *P. falciparum* infection. Infections in further pregnancies are usually less severe due to previous contact with VAR2CSA-expressing *P. falciparum* strains and increasing immunity to VAR2CSA (8). By accumulation in the placenta, *P. falciparum* also evades elimination processes in the spleen. In endemic regions, the peripheral infection can be controlled mostly with acquired partial immunity against *P. falciparum*, while the plasmodia may persist unrecognized in the placenta and can cause maternal anemia as well as fetal developmental disorders (9). In addition to *P. falciparum, P. vivax* can also lead to pregnancy complications. However, the consequences are generally less severe and are not presented in this review (4, 10).

Various measures and antimalarials can be taken to prevent and treat malaria during pregnancy but there is a lack of information regarding their safety, efficacy and pharmacokinetics. In all regions affected by malaria, early diagnosis and treatment as well as the use of ITNs (insecticide-treated nets) are crucial. In regions with endemic malaria, intermittent preventive treatment (IPTp) starting at the second trimester with the antimalarial drug sulfadoxine-pyrimethamine is additionally recommended for pregnant women by the World Health Organization (11). A metadata analysis confirmed that this therapy reduces the risk of low birth weight (LBW) when 3 or more doses are administrated, compared to the standard 2-doses regimen (12), but among the estimated 35 million pregnant women eligible for IPTp therapy, in 2017, <50% received two, and only ~22% received three or more doses of IPTp (11). Further efforts are needed to improve the coverage and access to IPTp for this vulnerable population.

Currently, the most effective first-line treatment recommended by the WHO for the general population is an artemisin-based combination therapy (11). This therapy resulted embryotoxic in animal studies. Therefore, less efficient and less well-tolerated medicines such us quinine and clindamycin have been recommended for women in first trimester pregnancy (13). However, the embryo exposure and toxic effects to artemisins may be different or lower in humans due to their specific placenta morphology (14). This may be supported by growing evidence that artemisins in first trimester pregnancy do not increase the risk of miscarriage, stillbirth or malformations when compared to quinine-based treatment (13, 14). For these reasons a clear conclusion of the risk/benefit ratio of antimalarials medicines cannot be drawn until further studies on pharmacokinetics and safety in humans will be conducted.

Finally, an additional approach to prevent malaria is based on current studies focused on the identification of the most immunogenic epitopes of the VAR2CSA antigen for vaccine development against placental malaria in pregnancy (see below) (15).

## *P. falciparum* Infection in Pregnancy

It has been estimated that, in 2007, out of 85.3 million pregnant women in areas at risk for *P. falciparum* malaria, about 2/3 lived in regions with stable (endemic) malaria (16). In these areas, one out of four women at delivery had evidence of placental infection (4). In sub-Saharan Africa alone, malaria was responsible for more than 10,000 maternal and 200,000 perinatal deaths annually until 2009 (2). In 2015, it has been estimated that 900,000 newborns suffer from reduced birth weight due to placental malaria (17). The exact incidence of *P. falciparum* malaria in pregnancy and the resulting IUGR cases remain unknown.

During pregnancy, the clinical appearance of a *P. falciparum* infection depends largely on the maternal immune status, which in turn is associated with the geographical region. If the woman has already acquired immunity, which is usually the case in areas with stable malaria, the infection is often asymptomatic. High fever and complications such as cerebral malaria, hypoglycemia, or pulmonary edema are rare. However, especially in their first pregnancy, women are not protected against placental infection. As the infection may persist undetected, it can lead to pronounced maternal anemia and serious consequences for the unborn child.

Low birth weight (defined as birth weight <2,500 g) is the largest risk factor for infant and child mortality in Africa (18). Independently of malaria infection, growth restricted babies have nine times higher risk of dying within the first month of life than normal weight newborns (19). Annually, around 100,000 children die in Africa as result of malaria-associated LBW (19). The major causes are premature birth and IUGR (20).

It is still unclear which period of infection during pregnancy is the most detrimental for fetal growth. In a recent study including 1190 pregnant women in Burkina Faso, maternal infection after the sixth month of pregnancy was significantly related to higher risk of LBW, while only a trend was found between early infection (≤4 months of pregnancy) and LBW (21). Conversely, a second study reported higher risk of LBW after infection in the second compared to third trimester or at delivery (22). And in a Benin cohort, only infection in early pregnancy was associated with LBW and maternal anemia at delivery (23). Remarkably, a high number of infections during pregnancy leads to an increased risk of LBW independently of the timing (22, 24). As the maternal immunological reactions change during pregnancy, more studies are needed to fully understand the interaction between infection and host response depending on the stage of pregnancy, and how this impare fetal growth.

Since severe maternal anemia and placental malaria are the major mechanisms responsible for malaria-related IUGR, they will be comprehensively addressed in the following sections.

### Maternal Anemia in Malaria

Severe anemia (defined as hemoglobin concentration Hb <7 g/dl) is the main complication of maternal *P. falciparum* infections in regions with stable malaria. In the sub-Saharan region, between 200,000 and 500,000 pregnant women are estimated to develop malaria-associated severe anemia (25–27). Severe anemia can lead to death even if only slight blood loss occurs during delivery. Circulation problems with an increased risk of heart failure and pulmonary edema may also occur (2, 9). In addition to malaria, other causes such as iron, vitamins (e.g., folate, vitamin A, and vitamin B-12) and trace element deficiency or worm infections may further contribute to the occurrence of anemia (28–30).

**Figure 1:**
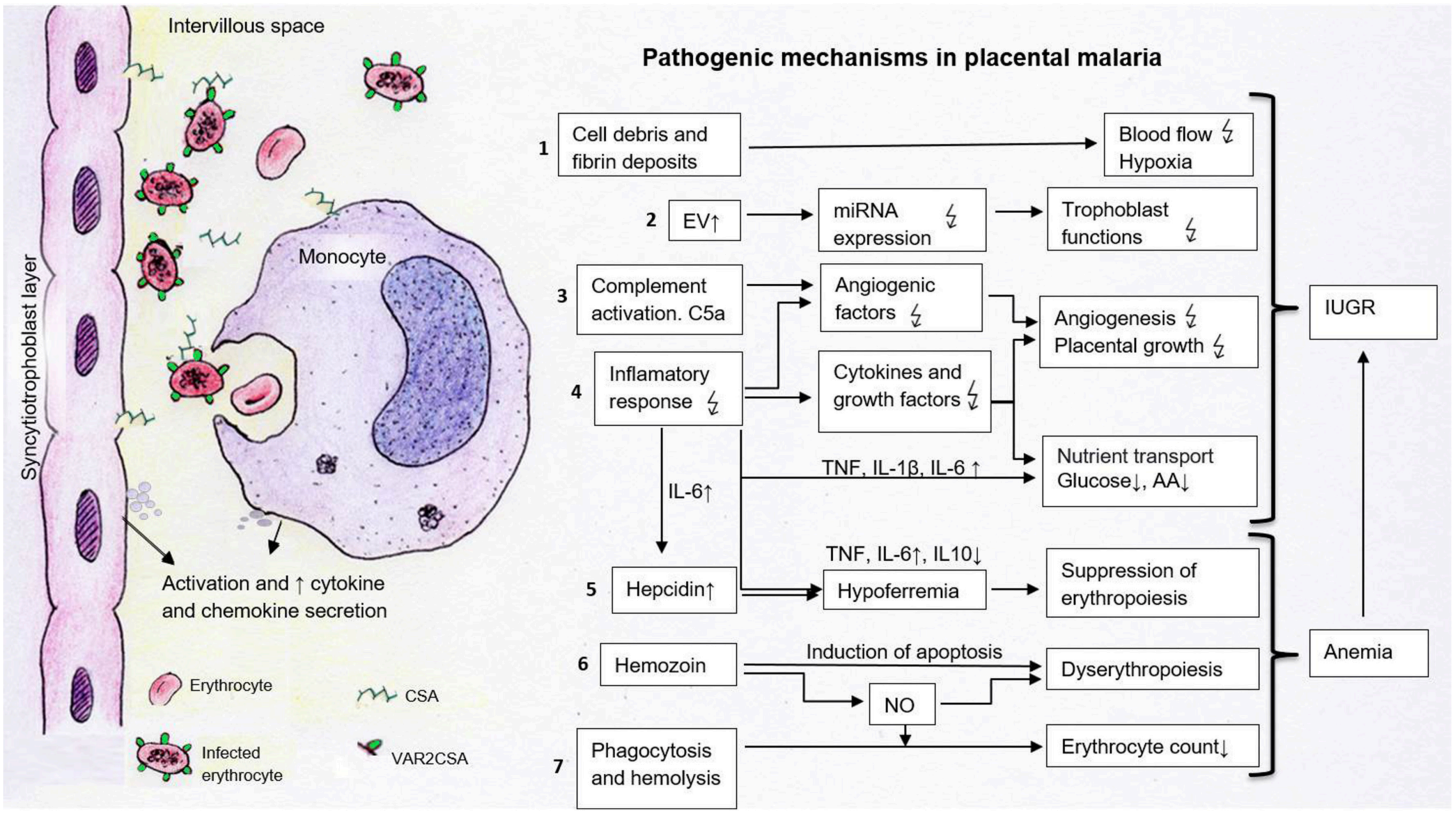
Pathogenetic processes potentially contributing to the development of IUGR in placental malaria. The sequestration of infected erythrocytes expressing VAR2CSA in the intervillous space occurs through the binding to placental chondroitin sulfate (CSA). This process leads to the activation of syncytiotrophoblast cells and local monocytes. These cells secrete chemokines and cytokines, which attract further immune cells which contribute to the pathogenesis of fetal growth restriction. 1. Cell debris and fibrin deposits can disrupt placental blood flow and lead to hypoxia. 2. Elevated EV may carry modified patterns of miRNAs disturbing trophoblast functions. 3–4. Activated complement factors (C5a) and inflammatory responses alter the concentration of cytokines, angiogenic and growth factors, affecting vascularization and placental growth. Cytokines and decreased growth factors impact placental nutrient transporters, resulting in decreased amino acid and glucose transport. 5. The suppressed erythropoiesis is caused either directly via cytokines or indirectly via increased hepatic hepcidin and lowered iron levels. 6. Dyserythropoiesis is a consequence of hemozoin deposits in the bone marrow, which disturb blood formation directly via apoptosis induction or indirectly via inflammatory mediators such as nitric oxide (NO). 7. Phagocytosis and hemolysis reduce erythrocyte numbers contributing to anemia. Together with placental alterations, maternal anemia leads to fetal growth restriction. 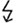, disturbed; ↑, increased; ↓, decreased; AA, amino acids; C5a, activated complement factor 5; CSA, chondroitin sulfate A, EV, extracellular vesicles; IL, interleukin; NO, nitric oxide; TNF, tumor necrosis factor.

The pathogenesis of anemia in malaria is generally multifactorial, even in non-pregnant women, and includes (I) hemolysis or phagocytosis of infected and non-infected erythrocytes, (II) disturbed development of erythrocytes from their precursor cells (dyserythropoiesis) due to hemozoin deposits in the bone marrow and (III) suppression of erythropoiesis as a result of a chronic inflammatory reaction (Figure 1) (31). (I) The elimination of non-infected erythrocytes is estimated to be almost 10 times higher than that of infected erythrocytes and thus plays also an important role in the development of anemia (32). Several potential reasons for this phenomenon are under discussion: hypersplenism and the increased activation of macrophages contribute to generally increased phagocytosis and cell lysis (33–36); non-infected erythrocytes also exhibit reduced deformability and are thus degraded in the spleen (37, 38); and the deposition of immune complexes and complement factors on non-infected erythrocytes that may cause receptor-mediated phagocytosis by macrophages (39–41).

(II) The malaria pigment hemozoin directly stimulates the apoptosis of erythrorid progenitor cells (42–44). *In vivo*, elevated plasma hemozoin concentration is associated with anemia and suppression of reticulocytes. The number of pigmented precursor cells in bone marrow correlates with the degree of abnormal erythrocyte development (43). Hemozoin also indirectly impairs erythropoiesis through inflammatory mediators such as TNF and nitric oxide (NO) from activated mononuclear cells (42). In *in vitro* experiments, NO synthesis in monocytes is stimulated by hemozoin. Increased nitric oxide production is associated with decreased hemoglobin levels in children with malaria anemia (45). In contrast, macrophages seem to protect the bone marrow from toxic effects of hemozoin (44).

(III) A further aspect in the development of malaria anemia is the suppression of erythropoiesis due to an inflammatory reaction. Malaria anemia is mainly caused by dysregulation of pro- and anti-inflammatory cytokines, chemokines, growth factors and effector molecules (31). Increased levels of TNF, IL-6 and IL-8 as well as decreased IL-10:TNF or TGF-β1:TNF ratios are associated with anemia (46–51). Proinflammatory cytokines also lead to hypoferremia with reduced hemopoiesis, a major mechanism of inflammatory anemia (52, 53). IL-6 stimulates the synthesis of the iron regulatory peptide hepcidin in the liver, which inhibits intestinal iron resorption and the release of iron from hepatocytes and reticuloendothelial cells (54, 55). In an experimental study on *P. falciparum* infection in five voluntarily participating adults, only slightly elevated levels of IL-6 and hepcidin were measured in serum, but a clear hypoferremia with strongly reduced hemoglobin concentration in the reticulocytes has been reported. The results suggest that the inflammatory disorder of iron hemostasis promotes the development of malaria anemia (56).

Several studies have described a link between maternal anemia and fetal development disorders (27, 29, 57). The reduced number of red blood cells (RBCs) together with altered properties of parasitized RBCs (PRBCs) result in deficient transport of oxygen and CO_2_ in the bloodstream. As consequences, chronic hypoxia and elevated oxidative stress arise in the maternal-fetal interface contributing to the occurrence of IUGR (58, 59) (Figure 1).

### Placental Malaria

Placental malaria is characterized by sequestration of peripheral erythrocytes in the intervillous space and the activation of immune responses leading to inflammation. The manifestation and extent of IUGR have been associated with the severity of placental damage promoted by *P. falciparum* infection. Numerous mechanisms are associated with placental dysfunction and IUGR, including inadequate trophoblast functions, disturbed transport of nutrients, morphological changes, and abnormal angiogenesis. In the following sections, the association between *P. falciparum* infection and these processes will be further discussed in the context of placental malaria. Figure 1 illustrates the pathogenic mechanisms of maternal anemia and placental malaria that contribute to IUGR.

## Structural, Cellular, and Molecular Mechanisms Linking Placental Malaria and IUGR

### Histopathological Changes in the Placenta

Decreased placental weight was detected in women with malaria infection and associated with the presence of placental inflammation and damage (60). In histological samples from patients with active *P. falciparum* infection, erythrocytes in the intervillous space are packed with parasites. In case of past infections, but especially in active chronic infection, the malaria pigment hemozoin can be detected in migrated monocytes or in fibrin deposits (61). Hemozoin deposits without parasitemia indicate an inactive infection (7, 62). Acute infection is more likely to be associated with preterm birth, whilst chronic infection is associated with maternal anemia and reduced birth weight due to IUGR (63). Further histological changes, especially in chronic inflammation, are fibrin deposition, clumping of syncytiotrophoblast cells, reduction of their microvilli, focal necrosis, and thickening of the trophoblast basal membrane (61, 62, 64, 65). These deposits can disturb blood flow in the placenta causing hypoxia and contributing to IUGR (Figure 1).

### Altered Inflammatory Response in the Placenta

Infection with *P. falciparum* leads to an inflammatory reaction in the placenta that shifts the balance between Th1 and Th2 immune responses toward the Th1 pathway leading to release of proinflammatory cytokines and migration of immune cells (5, 66). In dual *ex vivo* perfusion experiments on isolated human placental cotyledons *P. falciparum* induces upregulation of transcription factor c-fos gene expression and release of the macrophage migration inhibitory factor (MIF), whereas expression of other chemokines and proinflammatory cytokines has been discussed as unspecific responses to oxidative and hypoxic stress. The observed release of placental chemokines and cytokines has been directed toward the maternal compartment while only trace amounts have been detected in the fetal circuit (67). By sequestration of infected erythrocytes in the intervillous space, syncytiotrophoblast and local maternal immune cells express elevated levels of chemokines [e.g., macrophage-inflammatory protein 1 (MIP1), monocyte chemoattractant protein 1 (MCP1) and interferon-gamma induced protein 10 (IP10)], which cause an increased migration of monocytes (68–71). By means of phagocytosis, cytokine secretion and antigen presentation to T cells, they contribute strongly to the elimination of the pathogens (72). On the other hand, excessive monocyte infiltration contributes to malaria pathogenesis and correlates in numerous studies with negative consequences such as reduced birth weight, premature birth and maternal anemia (73–78). The role of natural killer cells (NK cells) in the placental immune response is controversially discussed. A complete absence of NK cells in the intervillous space of malaria infected placentas has been described, which may be partly responsible for reduced parasite elimination (78). Pregnant women with *P. falciparum* infection have significantly lower levels of INFγ-producing NK cells in the placenta than aparasitemic pregnant women (79). Moreover, the cytotoxicity of NK cells against infected erythrocytes is lower in primiparous women, who are particularly susceptible to malaria, than in multiparous mothers (80). These results suggest that a higher number of INFγ-producing NK cells protects against infection (79, 80). In another study, however, NK cells in infected placentas are elevated and associated with low HLA-G production in trophoblast cells, which may contribute to a negative pregnancy outcome (81).

Monocytes secrete cytokines for the differentiation and activation of further immune cells. The following proinflammatory cytokines can be found elevated in peripheral and/or placental blood of infected pregnant women: TNF (50, 51, 66, 71, 82–87), IFNγ (50, 51, 66, 71, 82, 83, 85, 88, 89), IL-1/β (66, 84, 86, 89), IL-2 (50, 82) and IL-6 (51, 66). In addition, elevated concentrations of antiinflammatory cytokines such as IL-4 and IL-10 have been detected (51, 66, 71, 82, 84, 85, 90). In some studies IL-2, IL-4, IL-6, IL-10, and TGFβ have been reported as decreased (50, 66, 86). Increased concentrations of TNF are associated with high placental parasite density and pregnancy complications such as LBW, IUGR, preterm birth and maternal anemia (50, 86, 87, 91). In one study, elevated IFNγ has been associated with reduced birth weight (50), while another study did not find this correlation (87) and a further study has shown that reduced IFNγ is associated with reduced birth weight (90). Other authors have found elevated IFNγ in non-infected multiparous women which is associated with the absence of infection and conclude an important role of IFNγ in protecting against infection (85, 89).

Increased IL-10 levels are associated with parasitemia, reduced birth weight and premature birth. The authors assume that IL-10 impedes adequate control of the pathogen (91, 92). In contrast, other authors claim that high IL-10 levels are important to counteract an excessive cellular inflammatory response and to protect against negative pregnancy outcome (50, 66). Since in placental infections IL-10 is also highly elevated in peripheral blood, it has been discussed as a potential biomarker for the diagnosis of infection with *P. falciparum* in pregnancy (84, 90). Furthermore, elevated levels of IL-6 and IL-8 may be associated with high parasite density and anemia, IL-8 with intrauterine growth restriction and IL-7 with absence of infection (51, 86, 89). Lower expression of IL-5 is associated with reduced birth weight (90). Table 1 summarizes the most important associations of elevated cytokines caused by *P. falciparum* infection with clinical parameters.

**Table 1:** Associations of cytokines with clinical parameters in *P. falciparum* infections in pregnancy.

**Elevated cytokine**	**Association with**	**References**
TNF	High parasite density and monocyte infiltration	(50, 87)
	Low birth weight	(87)
	Premature birth	(91)
	Intrauterine growth restriction	(86)
	Maternal anemia	(50)
	Fetal death in mice	(93)
IFNγ	Low birth weight	(50)
	Fetal death in mice	(93)
	Absence of infection	(85, 89)
	No association with low birth weight	(87)
Low IFNγ	Correlation with low birth weight	(90)
IL-10	Parasitemia and low birth weight	(92)
	Premature birth	(71)
	Control of inflammation	(50, 66)
IL-8	High parasite density and low Hb	(51)
	Intrauterine growth retardation	(86)
IL-7	Absence of infection	(89)
IL-6	High parasite density and low Hb	(51)
Low IL-5	Correlation with low birth weight	(90)

Alterations in the levels of placental Th1 and Th2 cytokines and growth factors as a response to *P. falciparum* infection may result in a disbalance which potentially impacts placental functions contributing to IUGR (Figure 1).

### Disruption of Hormonal Balance

Several of the critical hormones in pregnancy are altered in malaria infections, such as the steroid hormones estrogen and progesterone. In addition to their effects on placental development and function, they contribute to the adaptation of the maternal immune system to pregnancy which is crucial for establishing maternal-fetal immunotolerance and prevention of fetal rejection (5, 94). Cortisol, which is secreted by the adrenal glands and associated with stress responses, also performs physiological functions on the placenta and suppresses immune responses (95, 96). The polypeptide hormone prolactin exerts a plethora of effects on maternal metabolism and immune system. It is expressed by the pituitary gland, decidua, myometrium, and immune cells (97).

In infection with *P. falciparum, the* steroid hormones estrogen and progesterone, prolactin and cortisol are altered (89, 98–100). In malaria, estrogen (estradiol, E2) has been found decreased in third trimester peripheral blood (100), and in another study increased in the placenta (89). In the latter study, low levels of estrogen are found especially in multiparous women and are associated with the absence of infection. Pregnancy-maintaining progesterone correlates positively with maternal Hb and child birth weight and is reduced in malaria infection (89). Prolactin has been described as either unchanged or decreased in infected pregnant women (98, 99). Being regulated by progesterone, the dropping of prolactin levels may be related to progesterone shortfall. The concentration of glucocorticoids such as cortisol is significantly increased and associated with high parasite loads (98, 99). Cortisol also correlates with a low number of NK cells and inhibits their cytotoxicity (80, 101).

Changes in the levels of endocrine hormones ocurring in women with malaria may contribute to impaired placental functions and, hence, IUGR. Likewise, hormones have also the potential to interfere with maternal immune responses against the parasite, which potentially influences placental and fetal complications (Figure 1). The consequences of the observed hormonal imbalances, however, are poorly understood, and require further investigations.

### Disturbed Angiogenesis and Placenta Development

Doppler sonography studies of *P. falciparum* infected pregnant women (32–35 weeks of pregnancy) show abnormal uteroplacental blood flow associated with preterm birth, LBW and perinatal death (102). The underlying pathogenetic processes are complex and not fully understood. Growth and vascularization of the placenta are regulated by various growth factors such as IGF and angiogenesis factors such as angiopoetin (ANG-1/-2), VEGF and its soluble receptor (sVEGFR1) (103). Altered expression of these factors can severely impair the development of the placenta and the fetus (Figure 1). In malaria infected mice, decreased levels of ANG-1, an increased ANG-2/ANG-1 ratio and growth disorders of the fetus have been described (104). Dysregulation of angiopoetin can also be detected in exposed primiparous women and is associated with reduced birth weight (104). Reduced levels of ANG-1 are also associated with various histopathological changes of the placenta in infected pregnant women in areas with low malaria transmission (105). The activation of the complement system, particularly the factor C5, seems to be significantly involved in the pathogenesis of disturbed angiogenesis. In mice, activated C5 (C5a) leads to an increased release of sVEGFR-1 from monocytes, which binds VEGF and makes it ineffective. Important growth stimuli for placental vessels are missing, as a result rejection and fetal growth restriction can occur (106, 107). In case-control studies of pregnant women from Kenya and Malawi, C5a is significantly elevated in placental malaria infection (108, 109). Increased levels of C5a are associated with altered angiogenesis parameters and with babies small for gestational age (109). Levels of IGF-1, an essential growth factor, are significantly reduced in infected pregnant women compared to non-infected ones. Decreased IGF-1 levels also correlate with decreased birth weight (110).

Malaria infection and pre-eclampsia or pregnancy-associated hypertension have some similarities (111). For instance, there is reduced placental perfusion in both pregnancy complications (102, 112). Biomarkers for pre-eclampsia, such as sVEGFR1 and soluble endoglin, are often elevated also in placental *P. falciparum* infection (113–115). An increased risk of hypertension in young primiparous women with chronic malaria infection has been described (114). A similar link between placental infection and pregnancy hypertension has been reported also in a hypoendemic region in Senegal (116). Malaria in pregnancy seems to contribute to the development of pre-eclampsia by placental inflammatory processes with increased cytokine secretion (114–116). Subsequently, preeclampsia constitutes a risk factor for fetal growth restriction (117).

### Disorders of Nutrient Transport

Several studies support the hypothesis that in malaria infection, dysregulation of placental nutrient transporters contributes to fetal growth restriction (Figure 1). System A transporter, one of the most important amino acid transporters in the placenta, is downregulated in malaria infection (118, 119). In several studies, a reduced function of this transporter has been associated with fetal growth disorders (120–122). Its activity is particularly reduced in placental inflammation with monocyte infiltrate (118). In placental malaria infection, inflammatory mediators inhibit essential signal transduction pathways (119). *In vitro* studies show that proinflammatory cytokines such as 1β, IL-6, and TNF lead to System A transporter dysregulation (123, 124). As described above, they are elevated in malaria infections and associated with reduced birth weight. In addition, growth factors such as IGF-1, which stimulate placental amino acid uptake, are reduced in *P. falciparum infections* (110, 125). In infected placentas, the expression of GLUT-1, a transporter important for basal glucose supply, is also downregulated (126, 127). The GLUT-1 expression at the basal membrane shows a positive correlation with birth weight and a strongly negative correlation with the density of the monocyte infiltrate. These results suggest that the inflammatory response in the intervillous space leads to fetal growth restriction due to impaired transplacental glucose transport (126). In general, increased TNF levels, decreased IGF-1 levels and placental hypoxia are associated with dysregulation of glucose transport (128–130).

### Extracellular Vesicles (EV) Containing microRNAs in Malaria

In recent years, extracellular vesicles (EV) have reached the spotlight of intercellular communication, particularly in the maternofetal relationship. Current studies demonstrate that placenta-derived EV are able, for instance, to modulate maternal immune cells (131), platelets (132), and vascular cells (133, 134).

EV are packed with non-coding RNAs, including microRNAs (miRNAs), mRNAs, proteins and lipids, which after internalization, influence the behavior of recipient cells. Several pathological conditions, including preeclampsia (131), IUGR (135), and malaria (136) have been associated with alterations of EV content and functions.

The human placenta possesses a unique profile of miRNA which is dynamically expressed to supply the specific needs of the respective gestational age (137). The study of miRNA expression patterns in placenta tissue has revealed dominant expression of oncogenic, angiogenic, and antiapoptotic miRNAs during the first trimester of pregnancy, whereas the third-trimester is characterized by prevailing expression of miRNAs related to cell differentiation and tumor suppression (138).

Despite the accumulating reports of miRNA expression in IUGR or small for gestational age (SGA) cases associated with preeclampsia (139–141), only few studies have investigated alteration in absence of preeclampsia. Interestingly, these publications suggest an important role of placenta-specific and placenta-associated miRNAs in the development of IUGR. For instance, a report on the placenta-associated miR-141, which is highly enriched in maternal plasma, has shown its overexpression in placentas complicated with IUGR and confirmed the miR-141 target pleiomorphic adenoma gene 1 (PLAG1), which may contribute to the development of this pathology (142). Likewise, seven members of the Chromosome 19 miRNA Cluster (C19MC) are downregulated in placentas from IUGR pregnancies (143).

C19MC is a placenta-specific cluster, low expressed at the beginning of pregnancy, but highly expressed at term (137). Members of C19MC have emerged as important players in the regulation of trophoblast invasion, proliferation and differentiation (144, 145), and more recently, in the immune response to viral infections in pregnancy (146, 147). It has been proposed that C19MC members confer antiviral immunity to trophoblast cells (148) but their role in defense to bacterial or parasite infections remains unknown.

Only a handful of studies have been carried out to investigate the changes in levels of circulating placental EV and miRNAs as response to malaria infection. Most of these studies have used animal models or had very low number of patients, and thus, have not been included in this review. To our knowledge a single report has been published analyzing expression of these and other placental-miRNAs in plasma EV of pregnant women with malaria (136). The concentration of total and placental-derived microparticles in plasma, characterized by the presence of Pregnancy-Specific Glycoprotein1 (PSG1) on their surface, remains unaltered in women with malaria and increased in those carrying HIV. Independently of malaria or HIV infection, women who delivered growth restricted neonates have higher quantities of both total and placental-derived EV. Furthermore, the level of miRNA-517c, a miRNA belonging to C19MC, is elevated in vesicles isolated from patients with malaria compared to uninfected controls (136). miRNA-517c is also associated with the development of preeclampsia and *in vitro* studies have demonstrated its involvement in decreasing trophoblast invasion and angiogenesis, as well as in increased production sFLT1 contributing to placenta dysfunction (149).

In human subjects, peripheral erythrocytes from malaria infected patients produce higher amounts of EV than from uninfected individuals. The severity of malaria infection and efficacy of anti-malaria therapies can be assessed based on plasma EV content (150, 151). A set of miRNAs is dysregulated during the blood stage of *P. falciparum* infection in adults compared to uninfected subjects. Four miRNAs: miR-1246, miR-6780b-5p, miR-3135b, and miR-6126 were reported as of great importance to malaria pathogenesis due to their involvement in multiple processes, such as cell defense response, immune response, TNF signaling pathway, and T cell receptor signaling pathway (152). A broader analysis of the consensus disease phenocode revealed a group of miRNAs commonly altered in a diverse spectrum of human diseases including autoimmune and infectious diseases. Eighty-eight percent of the miRNAs of the consensus set have the potential to target the principal components of the nuclear import and the inflammasome pathways including KPNA1, NLRP1 (NALP1), and NLRP3 (NALP3) genes. After being upregulated in malaria, these genes return to normal levels in PBMC from patients treated with chloroquine (153), a drug considered in the antimalaria drug policy of the WHO (154) and used for malaria prophylaxis during pregnancy due to its relatively high safety (155).

New miRNA functions have been explored regarding the infection and propagation of malaria. Several studies have demonstrated that *P. falciparum* lack miRNA sequences in its genome, presumably by absence of argonaute and dicer genes (156, 157). However, the presence of approximately 100 human miRNAs was detected within the parasites suggesting a unidirectional transfer (158). During the blood stage of *P. falciparum* malaria infection, uninfected erythrocytes enhance the release of EV which mainly target infected erythrocytes and the parasites therein. An *in vitro* study demonstrated that these EV contain hAgo2-miRNA complexes including those of miR-451 and miR-140, which after internalization by the parasites result in effective downregulation of the essential malaria antigen, PfEMP1 expression (159). This finding supports a report on erythrocytes of sickle cell anemia patients, showing enriched human miR-451 and let-7i in erythrocytes, which are transferred to parasites and target cAMP-dependent protein kinase PKA-R mRNA. This results in inhibition of *P. falciparum* blood stage development and contributes to malaria resistance (158). Monocytes, macrophages, and neutrophils also become activated after being exposed to peripheral erythrocyte EV, indicating their contribution to inflammatory processes taking place during malaria infection (160). These findings highlight the role of miRNAs in the innate resistance of erythrocytes to malaria infection as a host mechanism to minimize disease severity. The study of miRNA in placental infection is also worth to be pursued since the expression of miR-451 and other six placenta-associated miRNAs is altered in primary trophoblast cells exposed to hypoxia and in plasma of pregnant women with IUGR (161).

It is tempting to speculate that EVs produced by *P. falciparum*-infected cells or other cells affected during the infection (e.g., immune cells) as well as their secreted miRNAs may play a role in the pathogenesis of placental malaria and IUGR (Figure 1). However, the potential effects and biological mechanisms by which these EV may influence trophoblast cell functions constitute a yet unexplored field. Despite being still at its beginning, the study of EVs and miRNA expression during malaria may contribute to identify novel biomarkers, to understand host immunoregulation and to develop new vaccination and treatments strategies.

## Interaction Between VAR2CSA and CSA in *P. falciparum* Infection in the Placenta

The pathogenesis of pregnancy malaria is mainly due to the fact that erythrocytes infected with *P. falciparum* bind to receptors in the placenta and accumulate in the intervillous space (sequestration). According to current research, the interaction between the VAR2CSA protein, a special variant of PfEMP-1 on the surface of infected erythrocytes, and CSA in the intervillous space of the placenta is regarded as the most important binding (162).

Sequestration of infected erythrocytes in the placenta mediated specifically by VAR2CSA has been confirmed using dual side *ex vivo* placenta perfusion (67, 163). Only *P. falciparum* infected erythrocytes expressing VAR2CSA but not those binding to endothelial protein C receptor (EPCR) or lacking PfEMP1 disappear from the maternal circulation and accumulate in the perfused tissue, mostly in the intervillous space and to a less extend on the syncytiotrophoblast (163). Therefore, understanding the molecular and structural processes of VAR2CSA-CSA binding and the mechanisms to inhibit this interaction may offer new intervention options, in particular for vaccine development.

### Chondroitin Sulfate a (CSA): Function, Occurrence, and Structure

CSA is a glycosaminoglycan, a sugar chain made up of disaccharide units which, bound to a protein, forms a proteoglycan (164). In 1996, CSA was described as a target structure for the adhesion of *P. falciparum* infected erythrocytes in the placenta (165). It has been detected by immunohistochemistry in the intervillous space and to a lesser extent at the syncytiotrophoblastic layer. It has been discussed that CSA is produced by the fetus and secreted into the intervillous space (166). In placenta infected with *P. falciparum* significantly higher concentrations of CSA have been found (166, 167). Since it is assumed that proteoglycans are involved in the mobilization of cytokines, hormones and growth factors in tissues, an increased expression of CSA may negatively influence placental functions and promote the development of complications through increased adhesion of parasites (167–169).

The understanding of the exact molecular and structural composition of CSA is fundamental to analyze its interaction with VAR2CSA (164). In addition, it is important for the development of novel therapeutic approaches, such as chondroitin sulfate oligomers that bind to VAR2CSA with a higher affinity than CSA and thus impede the sequestration of infected erythrocytes in the placenta (170).

### The *var2csa* Gene in *P. falciparum* Infection

In the presence of a placenta, a *P. falciparum* subpopulation switches to the expression of the *var2csa* gene, one of 60 var genes of PfEMP-1 (171). In contrast to the other var genes, which differ greatly between the strains, *var2csa* is widely conserved (172–175). The *var2csa* gene is detected in the analysis of the genome of *P. falciparum* isolates from different regions in almost all strains (173, 175, 176). Of the 60 var genes, only three, including *var2csa*, seem to be expressed in all three examined *P. falciparum* strains (3D7, IT4, and HB3 strains).

In earlier studies, *var2csa* and other var genes, such as *var1csa* and *varcs2*, have been associated with the adhesion of infected erythrocytes in addition to *var2csa*, but this could not be confirmed in more recent studies (171, 177–180). However, studies investigating the genome and protein synthesis of placental parasites have revealed other highly regulated genes and proteins that might be indirectly involved in the pathogenesis of placental malaria (181–185). While the disruption of the *var2csa* gene leads to the loss of CSA binding capacity (179, 186), no other highly regulated var genes are directly related to placental adhesion (187).

### VAR2CSA Protein in *P. falciparum* Infection

**Figure 2:**
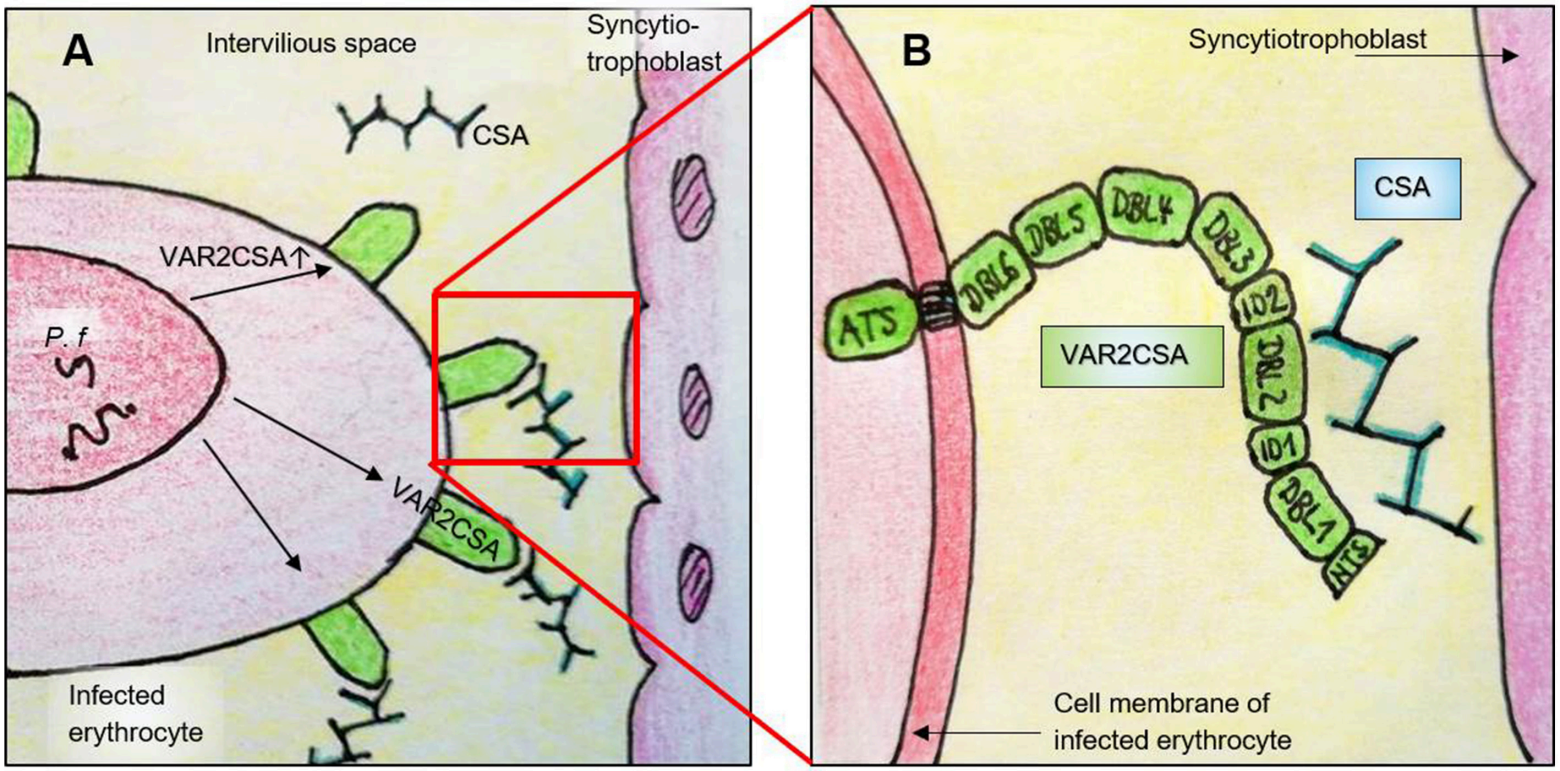
Simplified illustration of the interaction between VAR2CSA and CSA in the placenta. **(A)** *P. falciparum* expresses VAR2CSA on the surface of infected erythrocytes, which in turn binds to CSA in the intervillous space. Pf, *P. falciparum* within a parasitophorous vacuole; CSA, chondroitin sulfate A. **(B)** Enlarged view of **(A)**. VAR2CSA binds to CSA with its N-terminal domains. ATS, Acidic terminal segment; DBL, Duffy-binding-like domain; ID, Interdomain; NTS, N-terminal segment.

VAR2CSA is selectively expressed on the surface of *P. falciparum* infected erythrocytes in the placenta. It was discovered in 2003 by Salanti et al. and confirmed in numerous further studies as the most important ligand of CSA (175, 179, 180, 186–189). VAR2CSA is a large (350 kDa) transmembrane polypeptide. The extracellular part consists of six Duffy binding-like domains (three DBL domains each of class x and ε, omitted in the further text for reasons of clarity), a cysteine-rich interdomain (ID2a/b) and further short interdomain segments (ID1, ID4; Figure 2 and Table 2) (198). VAR2CSA is structurally and functionally very different from other PfEMP-1 proteins. For example, specific domains necessary for the recognition of vascular receptors such as CD36 and ICAM-1 are missing (162, 175). Rosetting—an otherwise important pathogenesis factor that describes accumulation of infected erythrocytes—is also atypical in placental malaria infections (199–201).

**Table 2:** Structure and sequence polymorphism of VAR2CSA domains relevant for CSA interaction and sequestration of *P. falciparum* in the placenta.

**Domain**	**Structure and sequence polymorphism**	**Strains in which domain binds to CSA**	**Strains in which multidomains bind to CSA**		
DBL1	7 VBs, 4 SCBs (190)	Does not bind to CSA			
Interdomain 1 (ID1)	Dimorphic region of 167 amino acid length; 78% variant (cluster 1) and 24% the second variant (cluster 2) (192)	Does not bind to CSA	3D7 (191)		
DBL2	8 VBs, 4 SCBs (190) Dimorphic structural motif of 26 amino acid length, which classifies two phylogenetic groups (the FCR3 and the 3D7 strain) (194)	3D7, FCR3		FCR3 (193)	FCR3 (195)
Interdomain 2 (ID2)	Not described	Not described			
DBL3	5 VBs, 4 SCBs (190) 3 variable sequences (V1-3) and four highly conserved sequences (C1-4) (196, 197)	3D7, FCR3			
DBL4	5 VBs, 4 SCBs (190)	Does not bind to CSA			
DBL5	7 VBs, 4 SCBs (190)	3D7			
DBL6	6 VBs, 4 SCBs (190)	3D7			

*VB, variable block; SCB, semi-conserved blocks*.

### The VAR2CSA Duffy Binding-Like Domains

Due to the size and complexity of VAR2CSA, only the quaternary structure of individual domains, but not that of the entire protein, has been described so far (202, 203). The best-known structure is that of the DBL3x domain (DBL3), which binds CSA *in vitro* and has been described in detail in two crystallographic studies (204, 205). DBL3 consists of an α helix with numerous inserted loops and can be divided into three subdomains. A loop between the second and third subdomain, which is disordered in the unbound state, assumes an organized structure in the presence of sulfate or disaccharides and forms a sulfate binding pocket (204). The conformational change creates a positively charged region that attracts the negatively charged CSA. It has been shown that mutations in these areas strongly affect the binding of CSA to DBL3 (205, 206). The flexible loop and other surrounding structures are located on the domain surface, are polymorphic and may protect the CSA binding site from recognition by the immune system (204).

VAR2CSA has numerous polymorphic areas compared to non-var proteins, leading to a high antigenic diversity and different placental *P. falciparum* strains (176, 207). Polymorphisms are important mechanisms for immune evasion and arise under selection pressure through exposure to the host immune system (176, 190, 192). They are mostly located on the protein surface, protect conserved areas from immune defense and hinder the formation of cross-strain antibodies (208–210). By analyzing the amino acid sequence of VAR2CSA and by comparing VAR2CSA sequences of different *P. falciparum* strains, conserved and polymorphic areas can be identified and the DBL domains of VAR2CSA can be further characterized. For instance, the DBL3 domain consists of four highly conserved sequences (C1-4) and three variable sequences (V1-3). Some of the conserved areas are also located on the protein surface and are target structures of naturally acquired antibodies (196, 197) (Table 2).

The sequence of the DBL2 region of VAR2CSA shows a so-called “dimorphic” structural motif of 26 amino acid length, which divides the strains into two phylogenetic groups (the FCR3 and the 3D7 strains) (207). Another dimorphic region of 167 amino acid length has been detected in the interdomain 1 (ID1), with 76% of placental isolates from Benin containing the first variant (cluster 1) and 24% the second variant (cluster 2). *P. falciparum* isolates with cluster 2 are associated with both multiple pregnancy and high parasitemia (192). The dimorphic areas seem to assume an essential function in pathogenesis, as they contain important elements for CSA binding and have remained stable for a long time in evolutionary history (192, 207).

The analysis of nearly full-length *var2csa* sequences from parasite isolates form around the world and also those reported at the GenBank and the *P. falciparum* genome sequencing projects (in total 106 *var2csa* sequences) revealed that the six DBL domains differ in amino acid conservation between 61 and 88% and are also structured into variable blocks (VB) and semi-conserved blocks (SCB, block B, D, F, and H) (190). DBL6 is the least conserved VAR2CSA domain with seven variable blocks consisting of a limited number of consensus sequences, i.e., similar or identical sequence patterns (211). Within DBL-6, the variable blocks 1 and 5 (VB1, VB5) from different parasite strains are recognized cross-reactively by antibodies from the plasma of exposed pregnant women (202, 212) (Table 2).

The gene diversity of *var2csa* is based on a high rate of self-recombination with a limited repertoire of sequences. In many variable regions the polymorphism is limited by already known or similar structural motifs (176, 190, 213, 214). As they may cause cross-reactive antibody production, these globally shared structural motifs as well as the conserved surface-exposed areas of VAR2CSA are of particular interest for the vaccine development (190, 196, 213). A summary of the structure and gene polymorphisms of individual DBL motifs are presented in Table 2.

### Interaction of VAR2CSA and CSA

To test if individual domains of VAR2CSA can independently bind to CSA, the DBL domains of two *P. falciparum* laboratory strains, the 3D7 and FCR3 strains, have been produced recombinantly and their binding to immobilized CSA has been measured *in vitro*. Four domains (DBL2, DBL3, DBL5, and DBL6) which bind to CSA have been found (Table 2). However, the results vary depending on the study and strain: from the 3D7 strain DBL2 (188, 215–218), DBL3 (196, 206, 216, 218), DBL5 (215, 218), and DBL6 (188, 206, 215, 217) and from the FCR3 strain, only DBL2 (188, 217) and DBL3 (188, 196, 205, 217) bind to CSA (Table 2).

Binding specificity and affinity of individual DBL domains also differ greatly from that of the entire extracellular section of VAR2CSA. Although some authors have demonstrated CSA-specific binding (188, 205, 216), the addition of CSC (chondroitin sulfate C) or HA (hyaluron sulfate) affects the binding to CSA of individual DBL domains but not of the entire VAR2CSA protein (217). Furthermore, DBL3 and DBL6 domains of the 3D7 strain bind nonspecifically various glycosaminoglycans, especially those with high sulfonation and many negative charges (206). Compared to the entire extracellular part of VAR2CSA, the affinity of the individual domains to CSA is up to 100,000 times lower. While concentrations of the entire protein in the nanomolar range are sufficient to bind >50% of CSA, micromolar concentrations are necessary for individual domains (191, 219, 220). These results suggest that individual domains do not have the same functional capacity as the entire VAR2CSA protein and that a specific and highly affine CSA binding requires multiple domains. This has been supported by studies demonstrating that combined domains in the N-terminal region of VAR2CSA can bind CSA with similar affinity as the whole protein (191, 196). According to Clausen et al., the minimal CSA binding region is located in the small ID1-DBL2b range, with DBL2b reaching up to 93 amino acids into the ID2a segment. Since ID1-DBL2 does not bind to CSA and ID1-DBL2b binds with high affinity, these 93 amino acids of ID2a appear to play an important role in the interaction with CSA. Although the ID1 region does not seem to be essential for direct binding, it is essential for the formation of a functional CSA binding protein (193). In two other studies, the core region of binding was found in the multidomains DBL1-DBL2/DBL3 (191) and DBL2-ID2b (195). Except for DBL2-ID2b and DBL1-ID2b all VAR2CSA fragments show specificity for CSA (193, 195). In summary, by combining several N-terminal domains around DBL2, a high CSA specificity and affinity can be achieved (Figure 2 and Table 2).

The spatial structure of VAR2CSA is very complex. In order to establish highly specific and affine binding to low sulfonated CSA, several domains of VAR2CSA appear to come into contact with each other and form a quaternary structure (210, 219, 220). This creates specific pockets, loops and structures that interact with the various functional groups of CSA (164). It can be assumed that VAR2CSA is an allosteric protein with positive cooperativity. This means that the binding of a functional CSA group to a domain of VAR2CSA changes the conformation of the protein in a way that the binding affinity for further CSA groups increases progressively (198).

### Further Relevant Binding Structures

It is under discussion whether other receptors besides CSA, including hyaluron sulfate (HA) and the Fc part of non-specific antibodies, contribute to the pathogenesis of pregnancy malaria (221, 222). Placental isolates can also bind to HA *in vitro* and at least some *P. falciparum* strains recognize both CSA and HA (221, 223). Placental isolates from Uganda show binding to CSA, HA and non-specific IgG and IgM (224). Further studies confirm the binding of the Fc part of IgM to VAR2CSA, which supports the hypothesis of immune evasion via unspecific blocking (200, 225–227). Other studies did not find significant binding of placenta isolates to HA or IgG antibodies (187, 219, 220, 228). Furthermore, HA does not appear in intervillous space, indicating that an essential role in pathogenesis is unlikely (167). As CSA is currently considered the main receptor for placental parasites, vaccine development focusses on inhibiting CSA-VAR2CSA interaction (193, 228). However, additional interactions, such as the immune evasion of parasites by shielding with IgM, can strongly influence the success of a vaccine or may lead to development of novel targets (163, 225).

### VAR2CSA for Vaccine Development

VAR2CSA is the leading candidate for a vaccine against malaria in pregnancy. Besides other preventive strategies the vaccine may be given to girls before puberty. This contact with the VAR2CSA protein leads to immunity against VAR2CSA-expressing *P. falciparum* which, in pregnancy, prevents the sequestration in the placenta (229).

Polymorphism and size (350 kDA) of VAR2CSA protein remain two major challenges that, thus far, have prevented the production of the entire protein for vaccination purposes (230). Therefore, the focus of current research is the identification of the minimal binding area within VAR2CSA, which has a high CSA binding affinity and specificity similar to that of the entire protein, and which simultaneously, induces broad binding-inhibitory antibody production (213).

As described before, this minimal binding region is located in the N-terminal region of VAR2CSA (191, 193, 195). Further studies have examined naturally acquired antibodies in multigravida in endemic regions (231–234) or the expression of antibodies against the VAR2CSA-CSA complex in laboratory animals and *in vitro* (195, 235–239). Two multidomains (ID1-ID2a, DBL1-DBL2) of VAR2CSA have been found as promising vaccine candidates (191, 193, 236, 238–240). They are part of two placental malaria vaccine projects, the PlacMalVac and PRIMALVAC project, which started phase I clinical trials in 2013 and 2016, respectively (241, 242). According to current information, PlacMalVac is safe, well-tolerated and a phase II clinical trial is under preparation (242, 243).

## Conclusions

*P. falciparum* infection in pregnancy leads to a specific involvement of the placenta where infected erythrocytes express the unique antigen VAR2CSA which binds to CSA in the intervillous space leading to their sequestration. This process induces an inflammatory reaction of the placenta that activates monocytes to switch the release pattern of soluble factors. The consequences are manifold and include disorders of erythropoiesis, angiogenesis, blood flow, and nutrient transport which together impact placental growth, and finally, fetal growth.

The knowledge on the specific VAR2CSA expression and its detailed structure and binding to placental CSA has led to the development of novel vaccine strategies which have a high potential to reduce *P. falciparum* induced pregnancy disorders and IUGR in the near future.

## Author Contributions

UM has coordinated the writing and has done revisions and corrections on the manuscript. JS has written most parts of the manuscript. RF and DM-P have added the chapter on EV and miRNA and have done several modifications of the text. HS has critically read and corrected the manuscript.

### Conflict of Interest Statement

The authors declare that the research was conducted in the absence of any commercial or financial relationships that could be construed as a potential conflict of interest.
